# Advancing artificial intelligence-assisted pre-screening for fragile X syndrome

**DOI:** 10.1186/s12911-022-01896-5

**Published:** 2022-06-10

**Authors:** Arezoo Movaghar, David Page, Murray Brilliant, Marsha Mailick

**Affiliations:** 1 Waisman Center, University of Wisconsin-Madison, 1500 Highland Avenue, Madison, WI 53705 USA; 2 Department of Biostatistics and Bioinformatics, Duke University, Durham, NC USA

**Keywords:** Fragile X syndrome, Electronic health records, Artificial intelligence

## Abstract

**Background:**

Fragile X syndrome (FXS), the most common inherited cause of intellectual disability and autism, is significantly underdiagnosed in the general population. Diagnosing FXS is challenging due to the heterogeneity of the condition, subtle physical characteristics at the time of birth and similarity of phenotypes to other conditions. The medical complexity of FXS underscores an urgent need to develop more efficient and effective screening methods to identify individuals with FXS. In this study, we evaluate the effectiveness of using artificial intelligence (AI) and electronic health records (EHRs) to accelerate FXS diagnosis.

**Methods:**

The EHRs of 2.1 million patients served by the University of Wisconsin Health System (UW Health) were the main data source for this retrospective study. UW Health includes patients from south central Wisconsin, with approximately 33 years (1988–2021) of digitized health data. We identified all participants who received a code for FXS in the form of International Classification of Diseases (ICD), Ninth or Tenth Revision (ICD9 = 759.83, ICD10 = Q99.2). Only individuals who received the FXS code on at least two occasions (“Rule of 2”) were classified as clinically diagnosed cases. To ensure the availability of sufficient data prior to clinical diagnosis to test the model, only individuals who were diagnosed after age 10 were included in the analysis. A supervised random forest classifier was used to create an AI-assisted pre-screening tool to identify cases with FXS, 5 years earlier than the time of clinical diagnosis based on their medical records. The area under receiver operating characteristic curve (AUROC) was reported. The AUROC shows the level of success in identification of cases and controls (AUROC = 1 represents perfect classification).

**Results:**

52 individuals were identified as target cases and matched with 5200 controls. AI-assisted pre-screening tool successfully identified cases with FXS, 5 years earlier than the time of clinical diagnosis with an AUROC of 0.717. A separate model trained and tested on UW Health cases achieved the AUROC of 0.798.

**Conclusions:**

This result shows the potential utility of our tool in accelerating FXS diagnosis in real clinical settings. Earlier diagnosis can lead to more timely intervention and access to services with the goal of improving patients’ health outcomes.

## Background

In recent years the application of AI in medicine has shown tremendous success [1]. Application of computational models within clinical practice guidelines can reduce the time required for diagnosis, decrease the cost of screening, and eliminate many factors slowing the diagnostic process [2–4]. AI research in the development of diagnostic tools has shown high levels of success for different conditions including cancer [5, 6], cardiovascular diseases [7], glaucoma [8], allergy [9], and others [10]. Some studies showed that it can improve care beyond current limits of clinical practice [11] by predicting future events that are usually not identified by physicians until after they happen.

Despite significant success in research, the application of AI in health care has remained mostly at the design and development stage [12]. Concerns about accountability, patients’ privacy, risk of bias, EHR infrastructure readiness, and regulatory barriers are among the main reasons for slow adoption of AI in clinics. Clinical workflows, user needs, trust, safety, and ethical implications must be considered in the design, development, and deployment of AI-assisted medicine [12]. The initial design of many AI systems is often limited to one patient population specific to one location and context [12]. To determine broader clinical utility, effectiveness and generalizability, it is necessary to evaluate and validate the algorithm on real-world independent data [13, 14]. Here we discuss the possibility of using AI for the identification of individuals with FXS, an underdiagnosed genetic condition [15] with substantial lifelong impact on health and well-being of patients and their families [16].

We have generated quantitative evidence of successful implementation of a pre-screening approach in two health care systems (the Marshfield Clinic and the University of Wisconsin Health System). In this paper, by way of background, first we discuss the importance of early diagnosis of FXS and its potential impact on patients’ outcome. Second, we focus on current diagnostic practices and the gap between implementation of professional recommendations and actual clinical practice. Next, we describe our AI-assisted pre-screening tool, developed using the EHRs of the Marshfield Clinic Health System [16]. Finally, we perform external validation by testing our AI model on the EHRs of a second independent patient population, UW Health, a health care system that includes longitudinal EHRs of more than 2 million people. UW Health does not significantly overlap in patient population or geography with the Marshfield Clinic.

### Importance of early diagnosis of FXS

FXS is the most prevalent inherited cause of intellectual disability and autism. The reported prevalence of FXS varies by race/ethnicity and geographical location [17] and is estimated to be as high as 87,000 in the United States and 1,400,000 worldwide [17–19]. FXS is not curable, and no approved pharmacological treatment is available for this syndrome, although many treatments are currently in the development phase. It is associated with a wide range of symptoms and co-occurring medical conditions, with variable expressivity and penetrance [16], including social anxiety [20], intellectual and learning disability [21], behavioral problems [22], attention-deficit/hyperactivity disorder [23], sleep difficulties [24], language deficits [25], motor problems [26], sensory integration challenges [27], seizures [26, 28], heart valve disorders [16, 19], endocrine and metabolic problems [16, 19], digestive issues and genitourinary disorders [16, 19]. Early behavioral intervention is beneficial in improvement of patients’ functional outcome [29–31].

This inherited genetic condition impacts multiple members and generations of a family. Family members might have the “premutation” of the gene which increases their risk for a wide range of medical conditions as well as having children with FXS. A study of families of children with FXS showed that 25 percent of these families had a second child with FXS before the first child received a clinical diagnosis [32, 33]. Premutation carriers are often diagnosed as the result of cascade testing after a family member is diagnosed with FXS. Therefore, the underdiagnosis or late diagnosis of FXS could also impact multiple generations in the family. In recent years, FXS clinics have been helpful in providing specialized medical services and genetic counseling to patients and their families. However, these clinics are not accessible to most potential patients. The prevalence of the syndrome and its significant impact on the health of patients and family members make diagnosis of FXS a public health priority [16, 18, 19, 26–35].

### Current state of diagnostic practice

Diagnosing FXS is challenging due to the clinical heterogeneity of the syndrome. It has no evident physical phenotype at birth and the phenotypic characteristics vary among patients [16, 19]. Additionally, the X-linked nature of FXS results in variation in clinical phenotypes between the sexes, with females often experiencing milder symptoms than males due to X-inactivation. Furthermore, the similarity of phenotypes with other conditions leads to misdiagnosis, causing additional challenges and delay in referral for genetic diagnosis.

Current approaches in identifying individuals with FXS are not efficient and guidelines for diagnosis are often not implemented. The American Academy of Pediatrics, the American College of Medical Genetics and Genomics, the American Academy of Neurology, and the Child Neurology Society provide clear guidelines recommending that any individual with developmental delay, intellectual disability, and autism of unknown cause, or other conditions suggestive of FXS should be tested [36–39]. However, a recent study of individuals with a confirmed diagnosis of autism showed that only 13.2% of participants were tested for FXS, highlighting a significant discrepancy between professional recommendations and clinical practice [40].

Offering cascade testing to family members of a diagnosed person is instrumental in identifying cases within the family, especially in individuals with milder symptoms [41]. However, this approach is also imperfect as it relies on the diagnosis of an affected person, disclosure of information within the family and understanding of genetic risk associated with the condition [42].

Another diagnostic strategy is screening for the subset of women who could pass FXS to their offspring (i.e., screening women with a premutation) [36]. The American College of Obstetricians and Gynecologists and the American College of Medical Genetics and Genomics recommend screening for women with a family history of fragile X-related disorders who are considering pregnancy or currently pregnant [36, 43]. However, many individuals with the premutation are not aware of a family history of the condition, as it is often undiagnosed or sometimes not disclosed within the family. Therefore, this approach is not effective in identifying most women at risk of having children with FXS.

A recent study performed by our team showed that a significant gap exists between the current estimated prevalence of the condition and the number of individuals actually diagnosed with FXS. Our study showed that at least 70 percent of cases do not receive referral for genetic testing and thus are not getting the proper diagnosis [15]. That study provided quantitative evidence of the urgent need to improve current approaches. There is an unmet need to develop new *pre-screening* practices that encompass the complexity of FXS and can detect potential cases without relying on information about family history or genetic testing.

### Initial development of an AI-assisted pre-screening model

Our team developed an AI-assisted pre-screening model which is able to identify FXS cases 5 years prior to the time of clinical diagnosis based only on patients’ prior medical history. The model was created using de-identified longitudinal EHRs collected from patients served by the Marshfield Clinic Health System [16]. The patients included in the EHR data were representative of the general population of patients living in northern, central, and western Wisconsin. Most of these patients live in rural areas and their overall socioeconomic status is lower than the national average [44]. The EHRs included an average of approximately 40 years (1979–2018) of medical data per participants. The goal was to “predict” a diagnosis of FXS 5 years before the clinical diagnosis was entered into the medical record using only other diagnostic codes that were previously entered into the EHRs. To minimize possible noise, other errors, and missing data in EHRs, the analysis was restricted to diagnostic codes that appeared at least twice for a given participant (Rule of 2), and that were observed in at least 5 individuals. These criteria ensured the presence of sufficient evidence of positive diagnosis and reduced the chance of misinterpreting rule-out tests [45]. Therefore, a comprehensive high-quality dataset was used for the construction of this model.

To develop the pre-screening tool, all individuals in the Marshfield Clinic EHRs clinically diagnosed with FXS (55 patients; 11 females and 44 males) were identified and 5500 sex-age matched controls (1:100 ratio) representative of the general population were randomly selected. A subset of cases who were diagnosed after age 10 and their matched controls were selected for the prediction analysis. This criterion was applied to ensure the availability of sufficient data prior to the diagnosis of FXS. A supervised machine learning approach called *random forest* [16, 46, 47] was employed to construct the model. Random forest is a non-linear classifier that is able to detect important multivariate interactions in the data and can find combinations of diagnostic codes that differentiate cases form controls [46]. To measure the success of classification, AUROC is reported [48]. The receiver operating characteristic (ROC) curve represents how well the model was able to correctly identify FXS cases and controls. ROC curve plots the false-positive rate versus the true-positive rate for every possible decision rule cutoff (threshold) between 0 and 1. An AUROC of 1.00 shows 100% success in classification meaning that classifier was able to successfully assign all of the cases to the correct class. An AUROC of 0.5 represents random classification. The resulting predictive model was able to identify FXS patients with an AUROC of 0.798 without relying on any genetic or familial data. Our next step, reported here for the first time, was to evaluate the performance of this model in a new unseen dataset, i.e., an external validation study.

## Methods

### Study population

For this external validation study, de-identified EHRs from 2,084,289 patients (1,018,259 males, 1,063,894 females, 2136 unknown) served by UW Health were mined. Although both the Marshfield Clinic and UW Health provide primary, secondary, and tertiary care with specialists in pediatrics, genetics, and neurology to patients residing in the State of Wisconsin, the two health care systems differ in many ways. They work independently and do not overlap geographically. UW Health has more than 80 locations and serves patients from south central Wisconsin, with an average of 33 years (1988–2021) of patient health data. It is a university system with the overall socioeconomic status of the patient population higher than national average [49]. Whereas the Marshfield Clinic uses a locally developed proprietary electronic medical records system, UW Health uses a medical records system developed and maintained by Epic. These differences enable us to evaluate the pre-screening model beyond system-specific diagnostic practices.

For the present analysis of UW Health EHRs, to eliminate the possibility of any selection bias, we identified all individuals who received the FXS code (ICD10 = Q99.2 or ICD9 = 759.83) on at least two occasions [16]. Cases were solely identified based on their medical records. We did not recruit patients for further genetic testing. All individuals without a diagnosis of FXS were considered as potential controls. As in the initial study, a subsample of UW Health participants who matched cases on age and sex with a ratio of 1 to 100 was randomly selected as the control group.

### Evaluation of the performance of the pre-screening tool: external validation

As in the initial development of the pre-screening tool in the Marshfield population, we again restricted the input variables to ICD codes that appeared at least twice for a given participant, and that were observed in at least 5 individuals. Only FXS cases diagnosed after age 10 (and matching controls) were included in the analysis. To evaluate the generalizability of the classifier across the two health care systems, we used the model trained on the Marshfield sample to identify FXS cases in the UW Health population. The AUROC was again used as the measure of the classifier’s success when applied to the UW Health data, and the Mann–Whitney–Wilcoxon test (Mann–Whitney U test) was used to measure whether the classifier performed significantly better than random (AUROC of random classification would equal to 0.5). We also created an independent model trained and tested on the UW-Heath sample and reported the performance of the ten-fold cross validated model. Furthermore, we created a timeline representing the order and median age of being diagnosed with key known conditions associated with FXS including speech and language disorders, developmental delay, attention deficit hyperactivity disorder, and intellectual disability.

## Results

### External validation on UW health population

87 participants (60 males and 27 females) were identified as having a clinical diagnosis of FXS (i.e., rule of 2) in the UW Health EHRs, with a median age of 30 (age range at the time data were extracted: 4–84 years) and the median age at diagnosis of 13 (range less than 1–84 years). There were no significant differences between cases from UW Health and Marshfield regarding age at the time of data extraction (p value = 0.88) and age of FXS diagnosis (p value = 0.70). To ensure the availability of sufficient data prior to the diagnosis of FXS, we created a predictive model focusing only on the individuals who received the diagnosis at age 10 or older. 52 UW Health FXS cases met this criterion (21 females and 31 males) and 5200 age-sex controls were selected for the analysis. 35 cases who were diagnosed before age 10 were not included in the analysis.

**Fig. 1:**
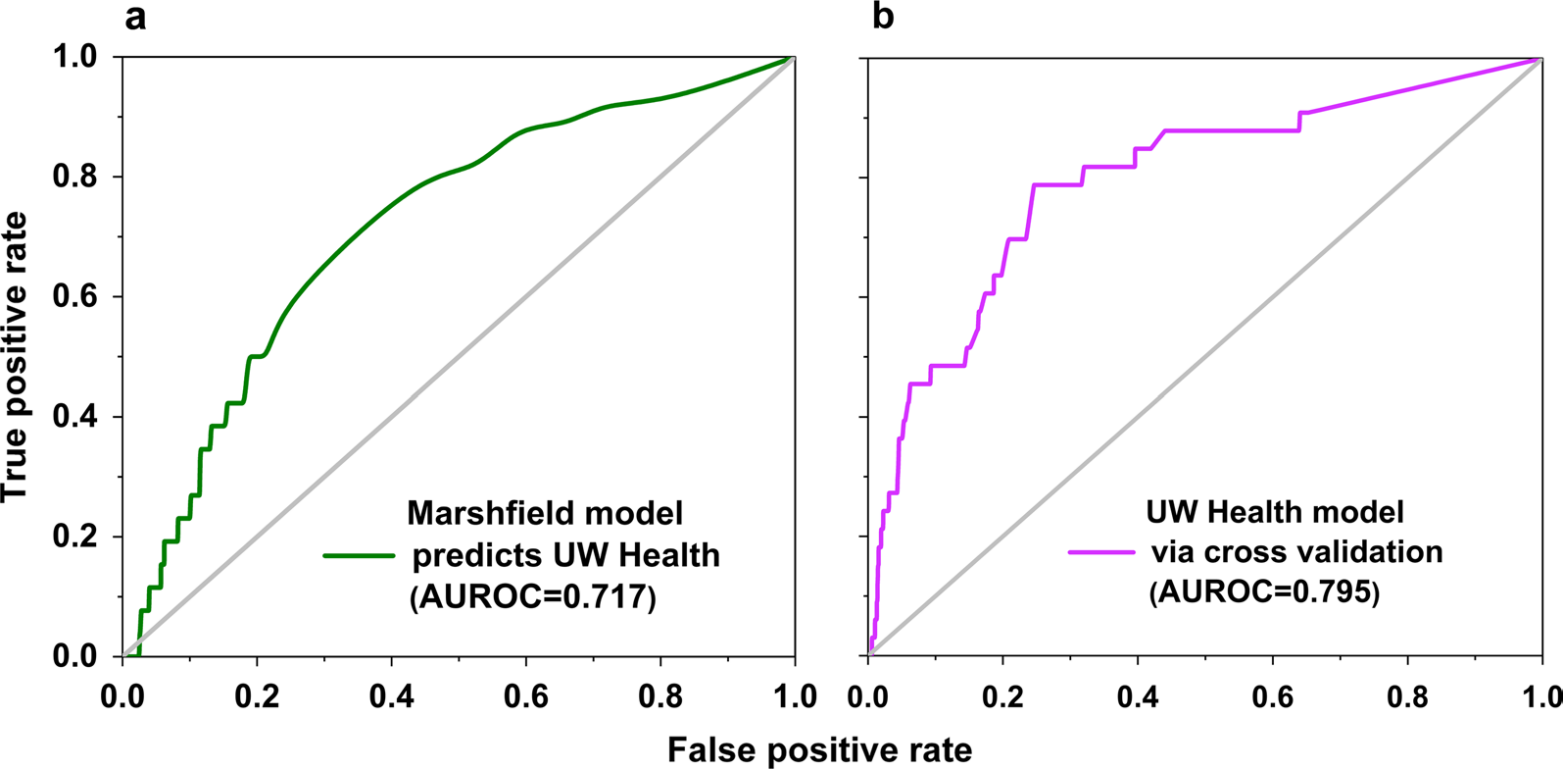
Artificial intelligence-assisted diagnosis. Receiver operating characteristic curve of classifier performances identifying individuals with FXS using their EHR data 5 years prior to receiving clinical diagnosis. Cases and controls are matched on sex and year of birth with 1:100 ratio. **a** Prediction of FXS status of UW Health subjects, using Marshfield model (AUROC = 0.717, p value = 2.9e−05), **b** Prediction of FXS status of UW Health subjects, cross-validated model (AUROC = 0.795, p value = 1.2e−09)

As shown in Fig. 1a, the model trained on the Marshfield sample successfully identified cases in the second independent health care system, i.e., UW Health, with AUROC = 0.717, p value = 2.9e−05. Additionally, for replication purposes, we developed an independent model analyzing EHR data only from the UW Health sample, using a ten-fold cross-validated random forest classifier. As shown in Fig. 1b, we were able to successfully identify cases from controls in this population, with AUROCs of 0.795 (p value = 1.20e−09). The performance of the replicated modeling approach on the UW Health population is almost identical to the performance of the initial model on the Marshfield Clinic population (AUROC = 0.798).

### Timeline of key co-occurring conditions

**Fig. 2:**
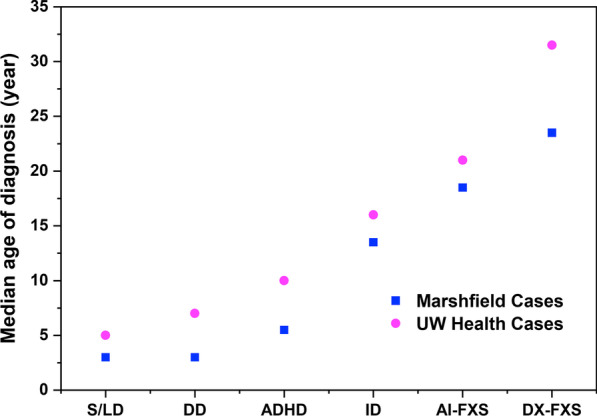
Timeline of median age of diagnosis for key conditions associated with FXS. *S/LD* speech and language disorders, *DD* developmental delay, *ADHD* attention deficit hyperactivity disorder, *ID* intellectual disability, AI-FXS, artificial intelligence assisted-prediction of FXS diagnosis, *DX-FXS* clinical diagnosis of FXS as reported in the medical report. Our AI-assisted approach is able to identify cases 5 years earlier than the time of clinical diagnosis

We created a timeline representing the order and median age of being diagnosed with key known conditions associated with FXS, based on these 52 cases in UW Health (Fig. 2). The overall timeline shows that these cases were diagnosed with developmental delay and speech/language disorder at a median age of 5, ADHD at age 7.5, anxiety disorder at 10, and intellectual disability at 16. However, they did not receive the FXS diagnosis until the median age of 31.5 years. A similar pattern was previously reported in the Marshfield population.

## Discussion

It is critical to provide sufficient evidence that new knowledge discovered in initial research is robust and reliable [50]. In this retrospective study, we validated the performance of an AI-assisted pre-screening tool in predicting FXS diagnosis using an independent population-based source of EHRs.

By incorporating a combination of co-occurring conditions, an AI-assisted pre-screening tool was developed and validated to identify potential cases at least 5 years earlier than the time of clinical diagnosis. The success of the AI-assisted pre-screening model on an independent set of new samples validates the generalizability of our approach and provides strong evidence of the possibility of using this approach in the identification of undiagnosed cases. All data used in this study are directly collected in a medical setting and are in fact real world data from actual patients, providing further proof of its potential utility in real world clinical applications. The AUROCs of the predictive models created and evaluated using the Marshfield cases and the UW Health cases were almost identical (0.798 vs. 0.795), representing the high level of reproducibility of results in different health care systems. The EHR systems in the two health care systems were completely different (Epic vs. a locally-developed electronic medical records system), further strengthening the validation and reproducibility of the modeling approach.

The two populations used for this research were comparable in terms of genetic background. Similarity of two populations reduces the systematic differences that potentially can confound the outcomes and negatively impact the interpretability of the results. Next, having provided sufficient evidence that the pre-screening approach is effective and robust, validation efforts should expand beyond these two health care systems to other populations, especially those from non-European ancestry [51–53].

Most previous studies of patients with FXS are based on a national volunteer survey of families of children with FXS and therefore do not fully represent adults, higher- functioning children, low-income families, families from diverse racial and ethnic groups, and others who do not volunteer for research. In this study, we included all individuals who received a diagnostic code for FXS. The socioeconomic status of patients in our research varies, with many of those served by the Marshfield Clinic being from low-income families. They were diagnosed at various stages of life, possibly due to different clinical circumstances (i.e., pediatric concerns, cascade testing and others). Therefore, our study is more representative of the general patient population.

The sex-age matched controls were randomly selected from the general population and there were no additional confounding effects compromising the outcome of the study. Therefore, the current study provides an independent unbiased evaluation of our AI-assisted pre-screening tool.

The pre-screening model is not intended to be a replacement for genetic testing, but it can serve as a tool to automatically alert physicians about the presence of multiple FXS-related phenotypes in the patient’s medical records. By prompting the physician to further evaluate such individuals and refer them for genetic testing and counseling, our approach could accelerate the diagnostic process and be instrumental in identifying un-diagnosed individuals in the population and addressing their health conditions.

The incorporation of our pre-screening model in the medical system would not require any changes in the current diagnostic workflow. We only used previously collected data and therefore no additional data collection would be needed. By accelerating the diagnosis, our approach could optimize the interaction between patients and physicians leading to provision of more timely treatment and care. Given the difficulty of implementing the professional recommendations for uniform screening, identification of potential cases who would benefit from prompt genetic testing is critical.

Furthermore, FXS testing is performed by a simple blood test and does not require any invasive procedures. Currently, in many cases, genetic testing for FXS is recommended as a rule-out test, and thus that a negative result can still be informative in patients’ diagnostic journey.

There are limitations to the current study that should be noted. Patients from both systems reside in the State of Wisconsin where the majority of the population is White (87.0%) [54]. Therefore, additional studies on more racially diverse populations are required as next steps to evaluate the generalizability of the findings. The case–control matching on age and sex with ratios representative of estimated prevalence of FXS [17, 18, 55–57] was not possible and in both studies a ratio of 1–100 was used to select controls. Additional studies on larger populations will provide more precise information on the performance of the model.

## Conclusions

Our AI-assisted pre-screening approach can facilitate and accelerate the clinical diagnosis of FXS and decrease the duration of the diagnostic odyssey and degree of stress experienced by patients and their families. The reproducibility of the results provides a high level of confidence in the potential positive impact of these findings, if incorporated in clinics and points of care. Our AI-assisted pre-screening tool could significantly improve the diagnostic process and could provide substantial benefits for patients, families and the health care system.

## Data Availability

The de-identified data that support the findings of this study are available from Marshfield Clinic and UW Health system, but restrictions apply to the availability of these data, which were used under license for the current study, and so are not publicly available. Data are however available from the authors upon reasonable request and with permission of Marshfield Clinic and UW Health system.

## Abbreviations

AUROC: Area under receiver operating characteristic curve
EHRs: Electronic health records
FXS: Fragile X syndrome
UW Health: University of Wisconsin Health System
